# Co-Creating the Cities of the Future

**DOI:** 10.3390/s16111971

**Published:** 2016-11-23

**Authors:** Verónica Gutiérrez, Evangelos Theodoridis, Georgios Mylonas, Fengrui Shi, Usman Adeel, Luis Diez, Dimitrios Amaxilatis, Johnny Choque, Guillem Camprodom, Julie McCann, Luis Muñoz

**Affiliations:** 1 Communications Department, University of Cantabria, Santander 39005, Spain; veronica@tlmat.unican.es (V.G.); ldiez@tlmat.unican.es (L.D.); jchoque@tlmat.unican.es (J.C.); 2 INTEL Labs Europe, London SW7 2AZ, UK; evangelos.theodoridis@gmail.com or evangelos.theodoridis@intel.com (E.T.); usman.adeel@intel.com (U.A.); 3 Computer Technology Institute and Press, “Diophantus”, Patras 26504, Greece; mylonasg@cti.gr (G.M.); amaxilat@cti.gr (D.A.); 4 Department Computing, Imperial College London, London SW7 2AZ, UK; fengrui.shi14@imperial.ac.uk (F.S.); jamm@imperial.ac.uk (J.M.); 5 Institute for Advanced Architecture of Catalonia (IAAC), Barcelona 08005, Spain; guillem@iaac.net

**Keywords:** co-creation, smart cities, IoT, platform, tools, experimentation, social innovation, OrganiCity, data observatory, opportunistic communications

## Abstract

In recent years, the evolution of urban environments, jointly with the progress of the Information and Communication sector, have enabled the rapid adoption of new solutions that contribute to the growth in popularity of Smart Cities. Currently, the majority of the world population lives in cities encouraging different stakeholders within these innovative ecosystems to seek new solutions guaranteeing the sustainability and efficiency of such complex environments. In this work, it is discussed how the experimentation with IoT technologies and other data sources form the cities can be utilized to co-create in the OrganiCity project, where key actors like citizens, researchers and other stakeholders shape smart city services and applications in a collaborative fashion. Furthermore, a novel architecture is proposed that enables this organic growth of the future cities, facilitating the experimentation that tailors the adoption of new technologies and services for a better quality of life, as well as agile and dynamic mechanisms for managing cities. In this work, the different components and enablers of the OrganiCity platform are presented and discussed in detail and include, among others, a portal to manage the experiment life cycle, an Urban Data Observatory to explore data assets, and an annotations component to indicate quality of data, with a particular focus on the city-scale opportunistic data collection service operating as an alternative to traditional communications.

## 1. Introduction

Recent studies [1] have predicted that, by 2050, 70% of the world population will live in urban areas, while more than a half of the world’s population already lives in cities. In this context, different stakeholders (city planners, politicians, researchers, etc.) implement policies that aim to improve the quality of life in urban environments, also developing initiatives contributing to more efficient and sustainable cities. The fact that cities represent a strategic meeting point between citizens and technology provides an additional dimension that can be exploited for a collaborative and continuous crowd-sourced creativity. This is what we categorize as societal innovation: human beings are immersed in a context which, based on Internet of Things (IoT) technologies, stimulates the conception of new ideas and solutions addressing the problems that are related to cities. To facilitate the adoption of such solutions, IoT experimentation under real conditions is crucial to their validation.

In recent years, experimentation with Future Internet (FI) technologies has been led by commercial companies and research centers, with less involvement from external stakeholders like citizens or decision makers. Due to the slow uptake, it is evident that a more holistic approach is needed, where all the relevant actors are involved. In this sense, IoT technologies are contributing to the creation of innovation ecosystems, where the FI provides an opportunity to the research community to modernize the existing solutions and adopt new ones, validated through the involvement of multiple stakeholders. Deployment, testing and evaluating solutions in cities under real conditions enables the possibility to conceive new or improve existing urban city services, such as waste or transportation management. Furthermore, the access to the vast amounts of urban data, generated from numerous sources, enables the design, implementation and ability to assess new techniques and algorithms that have the potential to outperform existing ones.

In this context, our work discusses how a novel approach designed and implemented in the OrganiCity project fosters the city’s growth and inspires societal innovation through a novel facility, through the encouragement of different stakeholders to utilize it, in order to co-create the cities of the future with new services and applications, and by improving current ones. It proposes the creation of a societal innovation laboratory underpinned by a distributed set of assets, such as the massive IoT infrastructure deployed in the city of Santander (Spain) or other data assets linked to the cities of London (UK) and Aarhus (Denmark), which allow experimental assessment of cutting-edge research on IoT-related fields and simultaneously support the provision of impact-generating smart city services directly perceivable by all stakeholders [2]. Last but not least, a collaborative experiment with different kinds of stakeholders is presented showing how the platform has been conceived with the required flexibility to accommodate different policies in terms of community involvement and also rewarding criteria.

This work is organized as follows: after a brief analysis regarding IoT experimental facilities and validation of new solutions for smart cities in Section 2, Section 3 begins with the presentation of the main requirements that provide the basis for the design of the facility. Then, the architecture of the OrganiCity facility is discussed, describing its co-creation tools and components, providing main insights on the Facility and Experimentation Management frameworks. In Section 4, the experiment life cycle is showcased with an actual example of how stakeholders can collaborate using opportunistic communications. By applying incentives, users can be rewarded for their participation in such experimentation systems. Section 5 provides initial conclusions along with future directions for this work.

## 2. Existing IoT Facilities at a Glance

Recently, experimentation with IoT related technologies in urban environments has attracted quite a lot of attention, especially in Europe. In this context, many research projects have been conceived to experiment with large scale infrastructures, developing different pilots that evaluate the proposed use-cases in the urban landscape. In such initiatives, the reader can find how different smart-city applications, outdoor deployments, and indoor installations in buildings targeting energy efficiency have been carried out. 

SmartSantander [3] is probably the most outstanding example of how a massive deployment of IoT devices is used within an IoT facility to allow experimenters to conduct research using innovative solutions in the city context. The experimentation of smart city architectures, services and applications in real-world urban environments has essentially been pioneered, deployed over a very large-scale IoT infrastructure in the city of Santander. 

Projects like IoT-Lab investigate crowdsourcing and IoT services for supporting multidisciplinary research tasks [4]. However, relatively little attention has been given to combining both an urban IoT infrastructure, comprising stationary and mobile IoT nodes, with a crowd-sensing component utilizing “transient” IoT resources contributed by citizens. The area of participatory sensing using crowdsourcing and harnessing ubiquitous technologies is discussed extensively in [5], where authors provide both the theoretical background and a review of a number of approaches currently utilized. 

However, although a number of technical advancements were made in these projects with respect to making available such IoT infrastructures to the research community and the industrial sector, there is still a lot of issues that need to be solved in a more coherent and holistic manner. The most relevant one can be considered as how to empower citizens or other stakeholders to participate in the societal change of the smart city innovation ecosystems. In this sense, the Experimentation-as-a-Service (EaaS) paradigm has been conceived to achieve this goal, although depending on the project, it is been implemented using different approaches. 

In [6], EaaS is a federated platform that provides reconfigurable on-demand access to a set of resources, allowing researchers to rapidly deploy experiments based on services belonging to different smart city domains. Although the user stands as the key actor of the experimentation, an integral framework and tools have not been defined allowing them to be part of the co-creation process. On the other hand, in [7] the EaaS concept is based on the creation of an Application Program Interface (API) that enables executing experiments over multiple existing IoT testbeds. In this case, the EaaS uses semantic-based technologies to provide an agnostic layer that enables federating the several IoT experimentation facilities. However, they have not designed the mechanisms to actively engage citizens in both defining application scenarios and participating in their conception, usage and therefore validation.

The implementation of EaaS solutions has recently grown in relevance, catching the attention of large companies and organizations. As an example, IBM (Armonk, NY, USA) provides an EaaS cloud-based platform that enables users to demonstrate and verify new products and technologies [8]. Also, in the city of Bristol (UK), a joint venture company provides a digital infrastructure that can be used by companies and developers to build and test a wide range of applications and smart city services [9].

Although SmartSantander also touched upon the subject [10], and the EveryAware project provides capabilities for environmental monitoring, data aggregation, and information presentation to users by means of mobile and web-based tools such as smartphones, computers and sensors [11], OrganiCity aims to provide some added value to these specific aspects, creating a novel facility that tackles the federation of different smart city platforms, assessing the crowd-sensing problem, and facilitating the experimentation of different stakeholders. The concept is even more ambitious because it is moving towards collaborative city making; citizens can identify urban challenges, co-create experiments to tackle these challenges, and advocate positive activism and behavior change [12].

## 3. A Novel Facility for Co-Creating

OrganiCity’s philosophy is that by empowering citizens, researchers and urban technology and service providers to jointly participate in a co-creation process, more effective and affordable smart city solutions can emerge faster, through a better exploitation of already-available resources and increasing local community insights. Such co-creation has the potential to rapidly provide the urgent answers cities of today are looking for in dealing with mounting socio-economic pressure [13]. 

Information and Communication Technologies (ICT) will continue to play an increasingly important role in how cities will be operated and transformed in the near future. However, the generation of new knowledge needs to follow the pace of data generation. To do so, one of the most pressing city challenges is to provide the mechanisms for knowledge generation to be more scalable and innovative, in a way that meets the increasingly complex challenges of the future city. In this sense, who knows the city better than those who live in it, walk its streets daily, make use of their services, etc.? This knowledge is a valuable source of ideas of what the city really needs and thus makes the citizen a key actor among the traditional stakeholders involved in the design of EaaS platforms. Because of this, OrganiCity proposes using the key resource implicit in population growth—the creativity of people.

Not only are citizens the consumers of the many existing digital services, data streams and interfaces that make up the digital layers within the urban landscape, they can also be a crucial element in producing the technologies and services of contemporary urban life, city management and societal development. Therefore, there is a need for a more holistic response to the commanding urban challenges, a response that calls for initiatives spanning city resources and stakeholders, from top-down strategies to bottom-up mobilization. These must operate in concert with each other, not as mutually exclusive alternatives, but as being complementary. This is one of the most important aspects that differentiate the approach proposed by OrganiCity from other EaaS platforms described in the previous section. 

In this sense, it will augment the integrated facility with novel tools that empower citizens to be part of the co-creation process at different stages of the urban technology design lifecycle and provide different means of their participatory engagement. The principle of co-creation will be applied to the facility itself, involving citizens and relevant stakeholders in the design of the facility use cases and requirements, its tools and even in the execution of their experiments on top of it.

Co-creation of knowledge is an unheralded, emergent opportunity; at the same time, what hampers the growth of cities is not only the untapped potential of more participatory systems, it is also the understanding of how to meet the different organizational, governmental, economic and cultural challenges of a successful ecosystem of urban co-creation. OrganiCity responds to the need to both experiment with the opportunities of co-created models of knowledge generation, as well as explore its implications.

Next, the work presents the important considerations that were taken into account for the design and implementation of the OrganiCity EaaS facility. Besides that, detailed information about its constituting elements that are employed to support the experimentation to be carried out by different experimenters are described.

### 3.1. Important Design Considerations

The key ambition of the architecture discussed in this work is to essentially make the creation and design of technologies and services for cities more inclusive for citizens and communities. In contrast to previous approaches, it aims to tackle the question of how smart cities can be organically grown from citizens and communities, instead of being engineered by the vision of researchers, the enterprise sector and city governments alone. Under this central principle, OrganiCity aims to build a firm technological foundation upon which this approach is implemented. This foundation is the creation of an EaaS facility for smart city infrastructures and urban services design, which empowers citizens and other societal actors to become an integral part of the societal innovation process. 

Currently, there is a plethora of smart city solutions and technologies, meaning that such a system translates into an integration of heterogeneous assets: IoT deployments using various platforms, open data platforms that are not already integrated into other smart city solutions, middleware for specific use-cases, etc. On top of that, there is the need to integrate Open Datastores, public city services’ APIs and legacy Utility systems (e.g., public transportation, waste management) and combine all of these with a federated approach. Due to this, an important design aspect for the OrganiCity EaaS facility is that its capabilities are based on and exposed through the federation of different cities and assets.

On top of all, this is a facility that aims to be interoperable with a set of innovative integrated tools for enabling the co-creation of urban ICT infrastructures, knowledge and services. These tools include an Experimenter Portal to manage the experiment life cycle, an Urban Data Observatory (UDO) to explore different data assets, an Urban Data Annotation component for the annotation of the data streams to indicate quality, a city-scale opportunistic data collection service operating as an alternative to traditional communications, and a service co-creation framework. The ultimate goal is to offer both a system providing useful services to citizens and local authorities, as well as to decrease the complexity of development, prototyping, and testing applications for researchers, corporations and all kinds of stakeholders involved in the smart cities. 

Another relevant aspect to consider is the enhancement of urban data streams, by means of validation, ranking and extraction of knowledge. Although today there exists a wealth of available smart city data, relatively little attention has been given to obtain information from such data; the design of OrganiCity caters for such aspects by integrating special Urban Data Annotation mechanisms to allow for both machine-learning produced annotations, but also provide ways for citizens to make their own contributions with respect to characterizing smart city data. Ranking and validation components in the design of the system aim to further enhance its capabilities.

Finally, complementing the aforementioned aspects, the EaaS framework is meant to enable communities of engaged citizens and relevant stakeholders to contribute to the co-creation of the facility and to subsequent experiments. Apart from the tools available to the developers and the interfaces available to end-users, in terms of system design, a defining feature is that the end-users to a certain extent can behave also as data producers and reviewers of the data. Due to this fact, incentives and rewarding mechanisms, Community Management, and the Experimenter Portal to enable monitoring and management of the EaaS process should all be interrelated.

### 3.2. OrganiCity Facility Architecture

This section describes OrganiCity (OC) facility architecture by presenting the key service functions and interfaces that are exposed to the different stakeholders in the smart cities. As outlined before, it aims to create an Experimentation-as-a-Service framework that supports co-creation along different cities and with the involvement of the citizen (*Organicitizens*). This term is utilized to call the users of the facility, aiming to encompass all of the categories of actors that can be found in the cities of the future.

**Figure 1:**
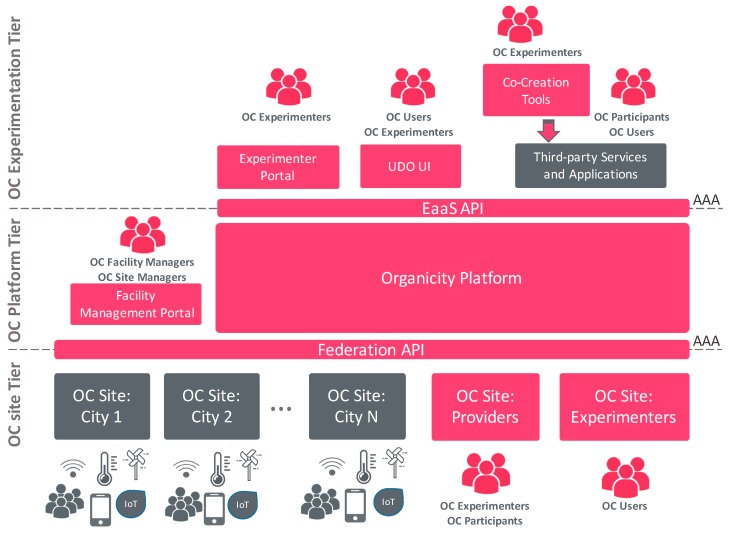
OrganiCity facility high level architecture.

To provide this EaaS framework, an architecture based on a three-tier level have been proposed. As it can be seen in Figure 1, the lower tier consists of Site infrastructures that represent federated urban data sources. More specifically, this tier consists of the city infrastructures (OC City Sites), the infrastructures and resources of experimenters (OC experimenters’ site) and the infrastructure supporting the generation of crowdsourcing data into the platform (OC Provider Site). The middle tier is represented by the OC platform and its components and, at the top level, there is the OrganiCity Experimentation tier, which facilitates the co-creation on top of the facility, as well as the administration components that deal with the configuration and management of the platform and its experiments.

Altogether, they form a powerful ecosystem, providing support for the co-creation and validation of a large set of smart city services and applications. One should have in mind that these components, to a certain extent, are meant to provide functionality to both advanced users/developers, as well as more “casual” end-users like citizens or even activists, decision makers or politicians. 

The facility provides an Authentication, Authorization and Accounting (AAA) framework that secures the interactions of different users and components with the OC platform. Through this mechanism, different users, depending on their roles, are able to interact with and within the platform. The different roles supported within the facility are Facility Managers, Site Managers, Experimenters, Participants or Users. 

Next, each tier is discussed in more detail, describing the key services and functionalities that each bring to the envisaged facility.

#### 3.2.1. OrganiCity Site Tier

The OrganiCity Site tier encapsulates services pertaining to an OC site under a common administrative ownership (e.g., a city sharing assets from IoT deployments, a company sharing assets, a citizen contributing data from his IoT devices, an experimenter creating assets during an experiment). OC sites consist of assets such as devices and/or datasets from open data platforms that a site aims to share with the users of the facility. To this end, the Federation API allows any user to integrate such data assets within the platform.

These assets may be heterogeneous in nature. In order for these assets to be accessible in the EaaS facility, within the OrganiCity Platform tier, a novel component called Urban Data Observatory (UDO) has been implemented. UDO services together with the UDO User Interface (UI) in the OC Experimentation tier facilitate the data discovery and browsing process to aid the development of experiments that relate to that data.

A city/site that wishes to make its devices or sources of information available for experimentation beyond mere data sources will require implementing custom experimentation (actuation, reprogramming) services towards these devices as an extension of the Federation API.

OrganiCity sites have specific accounts in order to be authenticated and authorized to share their assets with the facility. At this level, services hosted at the city infrastructures, can be used on Federation API to incorporate their data assets into the OrganiCity facility, being able to update their information accordingly whenever it is needed. Moreover, an OC site might implement site-specific management that encapsulates local management services, such as user management, device management, security framework, etc.

Aiming to support experimentation in the OrganiCity facility, a special component called OC Experimenters site has been conceived. It deals with the integration of data assets generated during concurrent experiments carried out by different experimenters of the facility. It relies on the AAA framework to guarantee the security and privacy of the data provided by different OC Users, IoT devices or other data sources. 

On the other hand, the OC Providers site support that any *Organicitizen* can contribute to the co-creation of the facility by feeding their own data in to the platform. To this end, it is required that they are registered in the platform as simple OC users. 

#### 3.2.2. OrganiCity Platform Tier

The OrganiCity Platform tier provides a federated view of federated assets used for experimentation across different OC sites and augments these with a set of innovative tools and services. A core component for the users is the Asset Discovery API within the UDO, which provides exploration and discovery services for time varying device centric urban data from IoT devices and Smartphones. Another key service function of the platform tier is a federated entity directory, called Asset directory, which combines exposed assets of all sites. This catalogue can be directly accessed via an Asset Discovery API or indirectly through UDO UI.

Facility Management (FM) framework allows the OC administrators to configure users in the platform along with their corresponding access rights. Besides that, the AAA framework ensures that all interactions with the platform are authenticated with valid user credentials and access rules for users with different roles, enforcing them over the different APIs and tools. The Facility Management Portal provides the means to manage the addition or removal of different cities infrastructures by adding or deleting OC sites.

Finally, it is important to highlight that advanced services and tools can experiment for experimentation through the EaaS API. This includes an Experimentation Management (EM) framework for handling and monitoring experiments, an Urban Data Annotation service and a Community Management service, including incentives and rewarding mechanisms for the participants of the experiments.

#### 3.2.3. OrganiCity Experimentation Tier

The OrganiCity Experimentation tier consists of various components (deployed services and applications, template applications or libraries) that facilitate the building of applications and services for experimentation with them. At this level, any *Organicitizen* with the experimenter role might develop and deploy its own services (e.g., a website, a web service, a desktop application or a smartphone application) that interact with the platform through the EaaS API and/or the Federation API. 

All applications at this tier have to be authorized and users utilizing them (either experimenters or participants) can be authenticated and authorized to interact with the various services/assets exposed by the platform. On this level, OrganiCity facility provides a set of user interfaces that aims to facilitate the experimentation and co-creation activities: UDO UI for discovering assets and the corresponding metadata associated to them like ranking and comments; Experimenter Portal for defining, managing and monitoring experiments during their life cycle; Web and Smartphone Applications or Services for gathering annotations or any other data provided by the users; and Community Management UI for interacting with the different communities of *Organicitizens* participating in each experiment.

### 3.3. OrganiCity Facility Components

**Figure 2:**
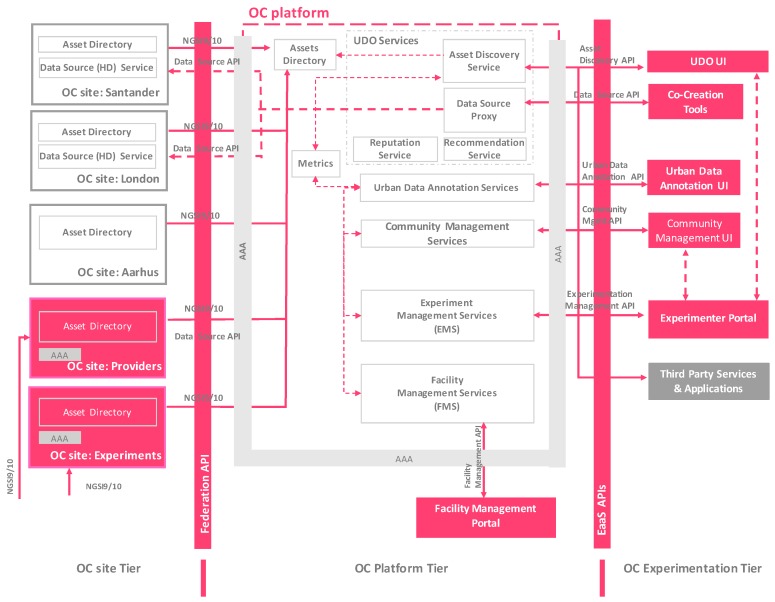
OrganiCity EaaS facility architecture instantiation.

This section presents a more detailed view of the OrganiCity paradigm, describing how the aforementioned components have been instantiated for building the integrated facility. The work focuses on the technical details of its constitutive elements, explaining aspects related to EaaS API, and the experimentation capabilities of the platform. Among others components, the Facility Management Services (FMS), the Experimentation Management Services (EMS) and rest of the basic elements enabling the experimentation in the facility are depicted in Figure 2 and outlined in the following sections.

#### 3.3.1. Facility Management Framework

This component deals with the configuration and management of the OrganiCity facility, providing a Facility Management Portal (FMP) as a graphical interface to OrganiCity administrators their daily labor and also the Facility Management Services (FMS) that facilitate the integration of this component with other ones within the platform. 

**Figure 3:**
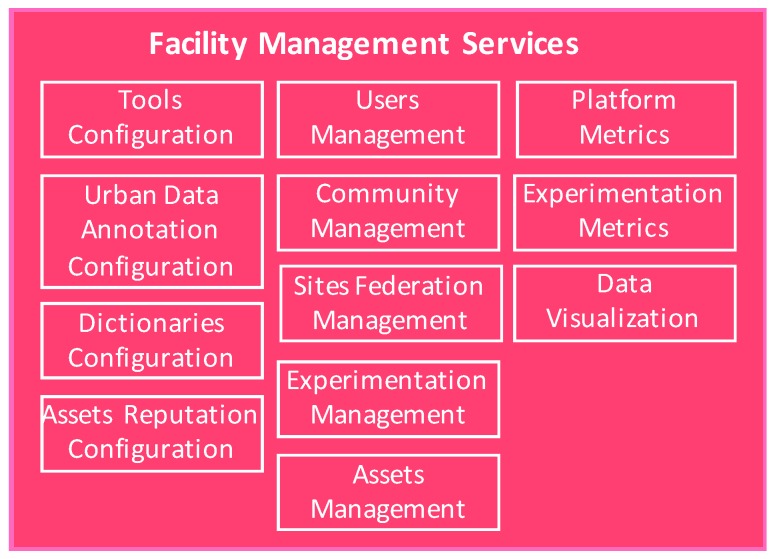
Facility management framework.

As can be seen in Figure 3, different elements within the platform can be configured:

- Co-creation tools. Registration and update of information about the co-creation tools that the experimenters can use within their experiments. This information is stored in an internal database, and it is made available to other components of the platform that may request it (i.e., the Experimenter portal visualizes the list of tools that are available in the left side of the screen).
- Applications types. They can be administrated from this tool, being able the manager to configure the types of application supported in the platform.
- Assets dictionaries. Definition of assets, attribute, units and data types that are used when the registration or updating urban resources into the platform. All these dictionaries, are consumed by the Asset Directory Service to present them to the experimenters or any other users of the OC facility.
- Urban Data Annotations. Within OrganiCity, tags for annotation of the quality of data assets are managed at two levels: facility level and experiment level.
- Reputation. Configuration the parameters that will permit to rank therefore the different data assets.

Moreover, OrganiCity administrators can manage different aspects of the platform:

- Users’ management: The OrganiCity facility allows to assign/revoke roles and permissions to the *Organicitizens* registered within the platform. To this end, the facility management component interacts with the AAA component. By default, when a user is registered within the platform then the plain OC user role is assigned to him/her. Using the management portal, the OrganiCity administrators are able to modify roles and permissions, based on the requests received and on detecting misusage of the systems.
- Communities’ management: Registered users in the facility can be to be organized in communities of users by both OC facility managers and experimenters. They are able to interact with these communities and communicate with them in order to promote the activities or their experiment/project, incentives and rewards.
- Sites Federation Management. Facility management portal allows OrganiCity administrators to add new sites and also to configure them (define the appropriate URL paths for the site services, create the proper subscriptions for streaming data updates, etc.). Besides that, OrganiCity site managers are able to configure the urban services that the assets belong to and see various visualizations of the data assets from a particular city.
- Experimentation Management. From the Facility Management Portal, it will be consumed data from the Experimentation Management Services (EMS) in order to visualize the experimentation activity within the OC platform. In case of detecting misuse within an experiment, the Facility Manager will be able to stop the activity of the experiment.
- Assets Management. Within the portal, there is a functionality that permits the OC Manager to visualize the data assets that are registered within the OC platform. Different intelligent searches have been implemented to facilitate the manager of the platform to see the activity, the annotations, reputation, etc.

Finally, the portal calculates in real time a set of metrics and Key Performance Indicators (KPIs) that present the activity within the platform, including dedicated insights about the experimentation that is taking place.

#### 3.3.2. Experimentation Management Framework

Another key aspect for building an EaaS facility is regarding the management of the entire life cycle experiments themselves, the components (i.e., applications, tools, and services) implemented and integrated with the facility under the scope of such experiments, and the results obtained throughout the duration of the experiment. The Experiment Management component deals with the administration of concurrent experiments running within the facility during their whole life cycle. It consists of a front-end, called an Experimenter Portal (EP), that allows experimenters to create and manage experiments, and also a backend service, the EMS that stores all the information related to the experiment in the internal repositories of the platform. This backend exposes a Representational State Transfer (REST) API that is used by all components of the platform that need to retrieve or update information related to the experiments. 

The EP implements a login and registration interface that allows experimenters to authenticate with the platform and access the experimentation dashboard. Among others functionalities, it offers the experimenter the ability to access the set of experimentation tools offered by OrganiCity, documentation and support channels. Additionally, experimenters can manage their already deployed experiments, like selecting data assets to be included, examining data assets that have been generated within the scope of a particular experiment, configuring and managing applications that are part of the experiment and monitoring the analysis of the results and metrics obtained during each experiment.

As the requirements for the experimentation provided by the different stakeholders are of different natures, in OrganiCity the Experimentation Management component has been designed and implemented, in order to homogenize the way that the different actors can manage their own experiment. Regardless, if they are experimenting with smartphones, testing Opportunistic Network scenarios or validating some services and/or applications on top of the OrganiCity facility, the EP UI is utilized in order to cope with all of those requirements. It provides a common interface to the experimenters to carry out this task, thus avoiding the burden of implementing ad hoc custom solutions for each experiment. 

**Figure 4:**
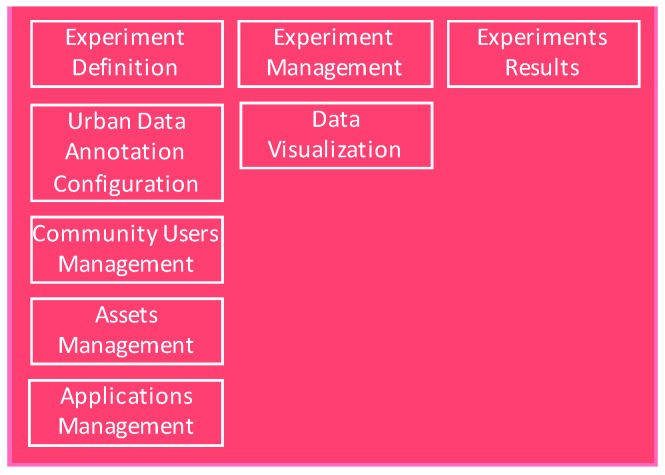
Experimentation Management framework.

As can be seen in Figure 4, different elements within the Experimentation Management framework can be highlighted. The EMS allows defining the scope of the experiment, providing spacious temporal configuration parameters, the tags to be used for annotating data during the experiment. During the experiment life cycle, one or various experimenters, dealing with the administration of a particular experiment, can get access to the set of assets that have been created in the experiment, the ones that have been selected (to consume data from them, mostly). Relying on the UDO UI, or the Asset Directory Service, the experimenter is able to visualize or retrieve such a list of data assets. Additionally, the applications that are part of the experiment can be administrated, providing specialized functionalities for the different types of experimentation supported in the platform. Finally, thanks to tracking activity carried out within each component, the experimenter is able to see in real time the results of the experiment. 

#### 3.3.3. Urban Data Observatory

The UDO is a set of services and UI tools that facilitate the ability to develop a deeper understanding of the federated city data sources that can be used by various types of stakeholders like data scientists, city decision makers, local organizations and citizens, among others. It is based on an Asset Directory (a data source repository) that maintains entries for the federated urban assets registered at the facility from the city sites. These entries contain contextual information about the data sources (e.g., a position inside the city, the type of urban observation, method of observation, unit of measurement, the urban service providing the asset information and various endpoints to acquire previous observations of the assets and possibly running aggregation queries on them), the latest state of the data sources (e.g., the last streamed values and date time of this update, etc.). The Asset Directory exposes resources in a uniform way, creating in that way a single facet of data sources from different cities and urban utility services towards experiments, online services and applications that are developed on top of the OrganiCity facility. 

The Asset Directory is implemented on top of the NGSI9/10 (https://forge.fiware.org/plugins/mediawiki/wiki/fiware/index.php/NGSI-9/NGSI-10_information_model) information model, proposing a minimal set or recommendations on naming schemes on asset IDs and types, as well as urban observation types. This repository model can support various types of data sources like mixed static and real-time urban and social data streams. This includes all sources of data in the networked city, formal and informal, digitally created from sensor inputs and IoT devices, or digitized ad hoc from non-digital and informal sources (manual input from users) and open data stores. 

**Figure 5:**
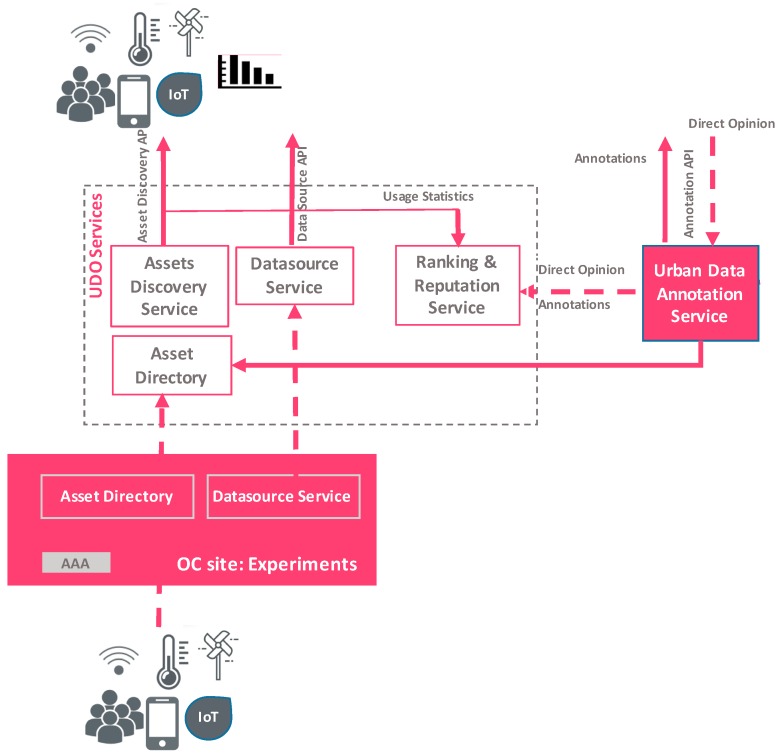
Urban Data Observatory architecture.

As can be seen in Figure 5, the Asset Directory, apart from registering and updating methods, is enhanced with a set of services that facilitate the discovery of urban asset, the exploration and experimentation with the data sources. The designed services, exposed through REST APIs, are:

- Asset Discovery Service: This service allows clients to list, explore and discover all the available federated resources of the OC platform.
- Datasource Service: This service allows access to stored data observation and previous states of the assets. This service actually acts as a proxy and delegates queries to corresponding endpoints of OrganiCity sites. In the case that an asset is not set up with a compatible datasource service, the clients’ custom interface accesses the provided endpoints.
- Ranking & Reputation Service: This service maintains the reputation and ranking scores for each federated urban asset.
- Annotation Data Annotation Service: This service handles various types of data annotations of urban assets.

Each one of the aforementioned services can be integrated into an experiment and the corresponding services and applications (websites, smartphone applications, etc.), or used in a stand-alone manner. Finally, urban data observatory is equipped with a visual exploration UI tool (http://observatory.organicity.eu), built on top of the UDO services, geared towards simplicity in navigation of the more complex underlying data sets and allow end-users to explore and combine the urban data to derive new insights about their city and community.

##### Visual Exploration and Discovery

The UDO expands the boundaries of traditional data platforms by providing features found in today’s top social and media sites. A special effort has been dedicated to the provision of a User Experience (UX) that feels familiar to advanced users. This primarily includes a rich map engine capable of dealing with big amounts of geo-located assets with complex geometries representations, a powerful text based search and a modular architecture for visualization modules. 

**Figure 6:**
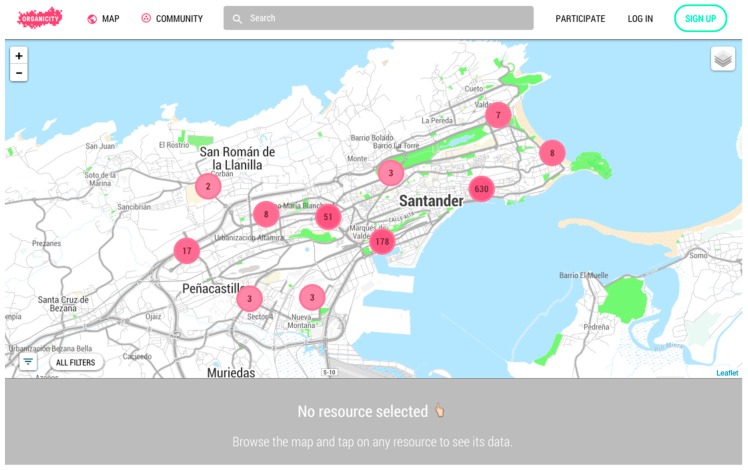
UDO geographic browser view.

The map module, illustrated in Figure 6, benefits from a tight combination of the front-end and the back-end architecture. This includes custom spatial methods provided by the Asset Discovery Service, together with a powerful web front engine. These features enable the spatial exploration of huge data collections, such as the ones found in smart cities, without hurting the end-user browser performance.

The second important functionality is text search, a key feature of the Asset Discovery Service, primarily focusing on incremental and contextual search. Incremental heuristic search is critical in providing users with fast feedback while they are looking forward for some specific data and keep tuning their search. However, the volume of data available within the platform requires this to be combined with contextual information, in order to support a more accurate sorting of the results. This includes information such as nearby assets or the user search history. 

**Figure 7:**
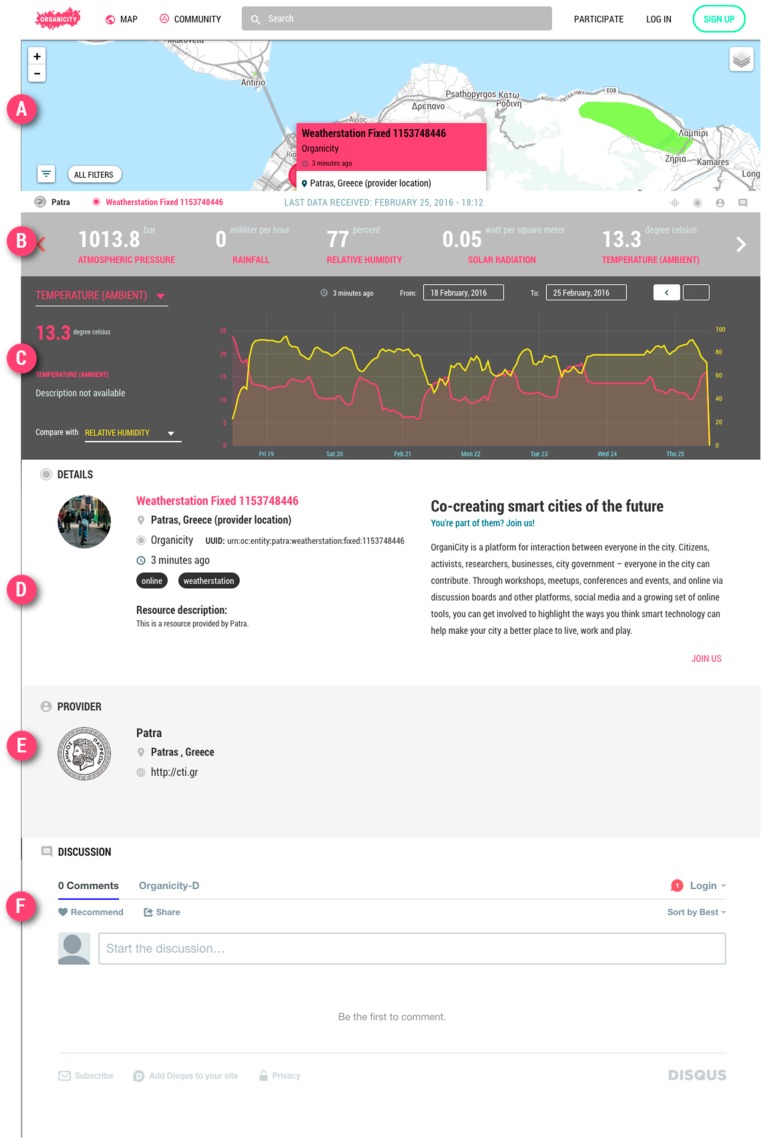
Assets visualization within the UDO UI: (**A**) Data Location; (**B**,**C**) Data Visualization; (**D**) Assets details and metadata; (**E**) Provider details; (**F**) Comments.

The last key feature is providing a modular approach towards integrating new browser visualization tools to support users in assets’ previewing and exploration. This is primarily achieved by the Model View Controller (MVC) front-end architecture, presented in Figure 7, decoupling the presentation from the data model and persistency layer. The current framework expands the boundaries of traditional libraries, supporting simply predefined chart types toward enabling novel visualizations to be developed in a modular and flexible manner.

##### Ranking of Urban Data Assets

As the UDO is federating heterogeneous resources from many cities and providers, data captured by various technologies/devices or manually. One may question whether “I can rely on these data?”, “Are these readings/observations timely and accurate?” These are quite important either during the selection of data sources, or with regard to conclusions you can extract from this data or when building your service/app on top of the urban assets and interacting with end users.

Building reputation mechanisms inside online systems is quite important and widespread, as large numbers of users interact online (by creating or consuming) with digital assets and services. As users might not have direct experience with digital assets, services and their providers, they usually base their decisions on reputations/ranking scores, where the experience of previous interactions from other users is captured. There is quite exhaustive literature in this field, tackling the problem of online services (see [14,15] for extended surveys on reputation systems and online services, and reputation models for web services, respectively). There is a wide set of options in terms of sources of information that can be used for modelling asset and user/community behavior and alternative modelling techniques: feedback only based models, statistical models, fuzzy-logic models, data mining models or game theoretic models. Moreover, data quality has attracted the interest as a large number of open data initiatives created around the globe focusing either on data-centric calculated metrics [16] or a more subjective user-centric approached [17]. In the field of IoT deployments and applications [18], more decentralized approaches are adopted for reputation modeling, utilizing face-to-face and location-aware interactions. 

The UDO models the reputation of the registered datasources by maintaining ranking scores. The underlying model integrates several qualities of the urban assets (similar to Quality of Service metrics), taking into consideration the preferences of consumers (as different users might be interested in different qualities or attributes), and finally having a subjective perception (e.g., what the users think about or rate asset qualities) and objective indications (try to evaluate asset qualities with calculated metrics). Reputation captures a combined measure of reliability inferred from feedback by a community of end-users. In our case, for modelling the reputation of urban assets, we rely on techniques for modelling reputation on online services, taking in mind at the same time that data sources might originate from IoT deployments, online data sharing APIs, open datastores and so forth. The reputation model is based on a statistical model that integrates both subjective and objective parameters of assets, built upon several sources of information such as:

- Usage Statistics: How many times an asset has been viewed or cited by OC users, how many times an asset has been used in experiments, etc.
- Direct Opinion of Users for Urban Assets: Direct opinions in the form of Like/Dislike, rating scores from users on three categories/groups of asset qualities like reliability (capturing how much reliable is the asset, taking in mind Correctness, Consistency, Precision and Accuracy and Completeness), availability (capturing how much available is the Asset taking in mind timelines, frequency of update and accessibility) and usability (capturing the overall effectiveness, efficiency and satisfaction on the usage of the asset);
- User’s credibility and preferences. Model reputation of an asset for a specific user might be affected by direct opinions of other users. During this calculation the model will give more weight (importance) to similar users or more credible users.

#### 3.3.4. Urban Data Annotations

Since the UDO is creating a new type of urban data repository and provides a starting point for exploration of urban data across different city environments, it is crucial to stimulate extraction and generation of knowledge from the raw data streams. Aiming at enhancing the urban data sources with useful information, OrganiCity has developed a set of services enabling collaborative Urban Data Annotation. In a way similar to the ranking of urban data sources, in order to integrate the active participation of citizens and their own opinions (also acquired and stored as annotations), a set of methods has been created for maintaining dynamic label categories, labels and labelling of data sources from the users. The utilized data model and annotation services are flexible enough to enable various types of labels from online resources on the Web, social media and references to rich multimedia content online (images, video, etc.) to free-text labels or numeric values. 

As described in the previous section, acquiring labels for a specific set of data sources can be parameterized under the scope of an experiment. Experimenters can define a set of label categories to be used by the applications associated with their experiment. Moreover, experimenters, or other end-users, can retrieve the various labels and the corresponding data under the scope of an experiment. Finally, they can create customized applications to acquire annotations from participants, or applications to visualize them. 

Acquiring a critical mass of annotations for constructing a useful knowledge base requires substantial contributions from end-users. For this purpose, the annotation process is integrated with the community management service (see the following section). It is possible for an experimenter to interact with the participants of the experiments, adjust the values or function of incentives given to them, as well as monitor progress in the annotation gathering process. Furthermore, it is possible at runtime of the experiment to adapt the used label set to extend, associate or abstract concepts represented through the tags (e.g., real-world events) and gather information that may be helpful for extracting further insights from the urban data sources. 

Along with the crowdsourcing annotation process, experimenters can utilize machine learning algorithms that enable more autonomous learning, semi-supervised learning or reinforcement learning techniques, exploiting the acquired annotations as training sets. It is possible in this way to use the created models as classifiers for automatic labelling of urban resources, events or anomaly detection. Furthermore, as users are constantly contributing with annotations, experimenters are possible to perform verification, cross-validation on the extracted models and create adaptive models using reinforcement learning methodologies.

The Urban Data Annotation services provide an API with basic methods for anomaly detection, a quite common task on IoT data streams. Experimenters, under the scope of an experiment, can train simple models by selecting resources and by providing a set of normal and abnormal samples. Then, the annotation service can produce a simple classifier that annotates updates of resources as normal/abnormal and store these observations as labels. 

However, other additional tools are required to simplify the extraction of insights and knowledge from urban datasources. Extensions to analyzing multiple streams IoT/metrics, user comments and discussions, social interactions and observations captured as multimedia are quite challenging and promising. Moreover, users carrying their smartphones can create annotations enhanced with user location, exact time, and contextual information (e.g., part of the city, inside tube etc.). This information it quite useful for producing more accurate and reliable urban observations). In this spirit, experiments for the interactive annotation of events, or sensed results, at the actual space and time of the event, or even by a group of users in a cooperative fashion, are quite important for understanding the state of the urban environment. 

#### 3.3.5. Community Management, Incentives and Rewards

As mentioned before, the EM framework deals with the administration of experiments. In some of them, it is necessary to establish mechanisms so as to promote its use in an intensive way, aiming to collect a large amount of data that in turn may be used to test it in an environment of stress in order to identify possible errors. This is the role of the Community Management tool, which enables the experimenter to populate its experiments among OrganiCity users whose profiles are related to the experiment objectives, so that a community can be defined for each experiment.

The community is created based on a set of users that is provided by another component of the OrganiCity platform, called User Management Service. This component stores the personal information that the user provides during the registration process, such as name, age, preferences on the subject of experiments that are tested, etc. Based on this profile, the experimenter selects users who might be interested in taking part in the experiment and sends the corresponding invitations.

Community Management enables the experimenter to both track users who downloaded the experiment and to understand the degree of its use. To do this, the Community Management system gets information from other components that log the user activity within the platform, for example, the annotations made in the data sources, the creation of new data sources, etc. Based on the level of user activity, the experimenter can define policies that encourage the user to make greater use of the application which includes setting rewards for users who reach the targets set in the incentive policies.

Community Management also enables the creation of communities with a broader scope than that offered by an experiment. In this case, the OC Manager, who has access to information of all experiments, will make use of the functionality offered by Community Management to establish strategies of incentive and rewarding. This enables the OC Manager to define communities involving users of different experiments to promote the relationship between them, establish templates communities that help experimenters to create their own, or create communities that promote user participation in activities that are carried out at the OrganiCity facility level.

It is worth highlighting that belonging to a community does not allocate any privileges for the user, so they are free to use any experiment without being invited to a particular community. The Community Management UI is a tool designed to help the experimenter, and the OC Facility Manager, to increase the use of experiments among potential users.

We provide advanced mechanisms through the Experimenter Portal (see also Section 4) in order to be able to monitor continuously in almost real-time the progress of an experiment executed through OrganiCity. These capabilities contribute to both the end users and the overall experimentation procedure in two interesting ways:

- Enabling to monitor in detail how experiments progress over time—By experiment progress, we refer to a set of goals set by experimenters with respect to each experiment submitted for execution. Such goals mostly revolve around spatiotemporal aspects of the data collected during the experiment.
- Using such components in order to implement incentive mechanisms that take as input the above information. This essentially translates into utilizing an algorithm which can have as input city areas or periods of time where the data collected is insufficient or of dubious quality, and use incentives to motivate crowdsourcing participants to collect the said data.

The latter aspect enables a whole new range of capabilities. Incentives in crowdsourcing is a popular subject in the field of crowdsourcing, with many studies examining incentive mechanisms and their application in existing systems [19,20,21,22]. We aim to utilize this existing body of work and incorporate such aspects in the way our system operates. By having such kinds of data available, it can then be fed as input to incentive schemes and algorithms, which can then decide how to distribute available crowdsourcing and other resources, in order to implement or advance certain tasks.

Consider the following example:

- While an experiment is being executed, the system checks upon the progress with respect to the area covered. At a certain point in time, only 50% of the designated experimentation area has been covered, while there are still available experimentation resources, e.g., people willing to participate and monetary awards.
- The system changes the current values of awards given for gathering data in the areas that have been already covered to zero, while it updates other, uncovered, areas with an elevated status in terms of awards.
- End-users, i.e., crowdsourcing participants, are notified of this change and adapt their behavior accordingly.

Expanding this concept, it is easy to imagine gamification extensions to such ideas, whereby introducing more “playful” mechanics to a crowdsourcing procedure, it becomes more engaging. The recent phenomenon of Pokemon Go attests to this notion, showcasing what is possible with such an approach.

**Figure 8:**
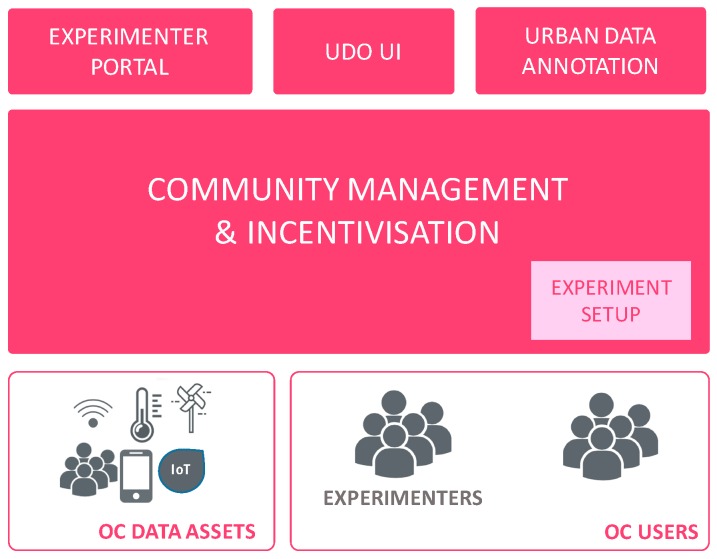
Interactions between various system components and the Community Management & Incentivisation component.

In terms of system architecture, Figure 8 shows how the Community Management and Incentivisation component has on the one side the available OC Data Assets and User Groups, and using the experiment setup and experimentation progress data (from Experimentation Management), it communicates with other system components such as the UDO and Smartphone Experimentation to relay information, or distribute crowdsourcing tasks in the case of the Data Annotation component.

In order to give an additional example of incentives that can potentially tie in with other system components, let us examine its utilization within DTN and the opportunistic networking paradigm described in the following sections. By design, delay-tolerant and opportunistic networking can easily integrate rewards in order to incentivize end users to participate. In such cases, where users essentially form the networking infrastructure, instead of employing a completely stochastic procedure and letting end users roam freely in urban areas, the incentivisation system could aid in “directing” users and channeling their mobilization into areas that affect the execution of an experiment or an ongoing urban IoT application.

OrganiCity does not check the “qualitative” aspects of the data collected for the time being; this aspect will be a subject in the future revisions of the system. Apart from incentive mechanisms, there are a number of end-user engagement elements embedded, e.g., in the smartphone experimentation platform, that provide certain feedback so that end users can monitor their own contributions. As a specific example, after end users’ contributions exceed a certain number, e.g., 2000 readings per day, they get a “badge” reward.

## 4. Engaging the Communities through the Experimentation Paradigm in the OrganiCity

As it has been presented previously, the OrganiCity facility provides the possibility that different stakeholders within the smart cities can co-create new services and innovative applications that can be tested and validated under real conditions in the urban landscape. In this section, it is explained how *Organicitizens* get benefit from the opportunistic communications framework that has been integrated within the OrganiCity facility, as it can be exploited in the smart cities’ use case [23], where IoT devices with extreme power constraints are utilized (e.g., static battery-powered IoT nodes may favor local-area over wide-area networking in order to conserve energy). 

Based on the fact that citizens spend several hours per day using their smartphones, they can potentially “collect” IoT data from other devices in the nearby area, acting as “data mules” for such devices. Additional restrictions, e.g., 4G data costs and caps, may translate into using multiple hops/smartphones to reach the upper layers of the opportunistic communication framework integrated within a facility. Essentially, this paradigm employs the ubiquity of smartphones to collect and relay data through Device-to-Device (D2D) communication channels, bridging the connectivity gap between different devices -ranging from Radio Frequency Identification (RFID) tags, sensors to smartphones with no data plan available- and virtual smart city infrastructures such as the one created through their federation into the OrganiCity facility.

To carry out such experimentation, an opportunistic communication middleware has been designed and implemented to be deployed in Android smartphones as Opportunistic Network (OppNet) Apps. Such applications collect data from external IoT devices (i.e., sensors) and relay it to upper OrganiCity layers through multi-hop opportunistic routing. Due to high energy and data costs for using cellular networks, the opportunistic communication middleware has been designed to uses only low-cost, short-range communication, such as WiFi and Bluetooth for data collection and to feed it to the facility, by means of the OC experimenters’ site. To this end, data is sent from the OppNet Apps relying on the Federation API exposed by the OC platform.

In order to provide incentives to the *Organicitizens* participating in the experiments, the framework takes into account economic aspects for its operation, by essentially letting participant devices trade data with each other in a virtual market. This trading of data is associated with “costs” (monetary or other type of rewards): the system essentially incentivizes the volunteers to participate in the process, as they can earn rewards by *selling* data to others and they are also willing to buy data from others, so they can sell it later at a higher price. Of course, this happens non-intrusively in the background of the phone and the users need not be aware of this ‘trading’ process. They only need to know what their periodic rewards are for participating in the data relay process.

**Figure 9:**
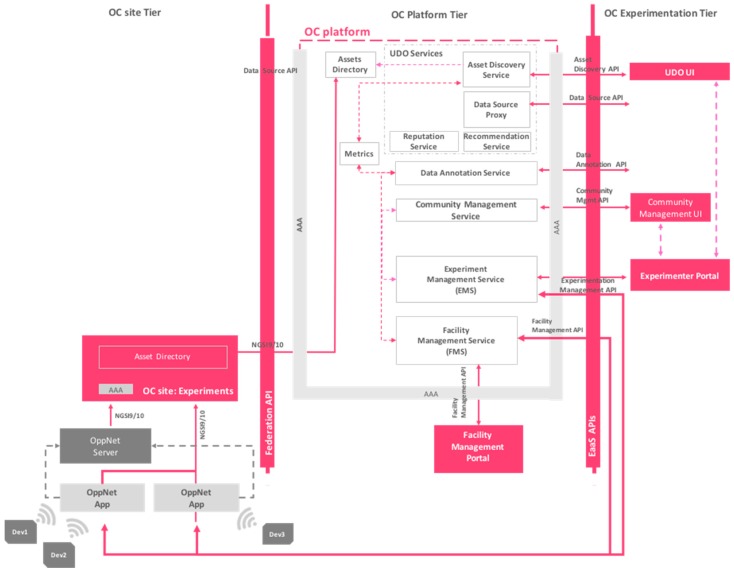
Overview of Opportunistic Communication integration with OC platform.

Figure 9 presents the integration of opportunistic communication framework and its components within the OrganiCity Facility. As it can be seen, the aforementioned three tiers can be identified. In the OC site tier, there is an OC experimenter site that federates data from concurrent experiments feeding data to the OC platform. In the case of opportunistic communication experiments, it gathers and processes all the sensor data sent from the OppNet applications to the OrganiCity. Moreover, there is an additional component called OppNet Server that keeps track of a set of related statistics that are useful for monitoring the execution of the whole experiment, e.g., delay, number of hops, readings produced/relayed, etc. This component calculates the rewards based on the incentives scheme defined and sends such information to the OrganiCity platform. At the OC platform tier, assets created and also statistics and data sent by the OppNet server are stored in the Asset Directory component. By means of the Asset Directory service of the UDO, users can explore and discover such assets. Finally, at OC Experimentation tier level, the experimenter uses the Experimenter Portal to see the information about the experiment, the relay nodes, and the end nodes and so on. Additionally, he/she will be able to see in real time the results of the experiment.

### 4.1. Opportunistic Communication Experimentation Management

For a better understanding of how experimentation is carried out in the OrganiCity facility, this section showcases the creation and execution of an opportunistic communication experiment during its life cycle, as the majority of the steps described here are common for all experiments.

**Figure 10:**
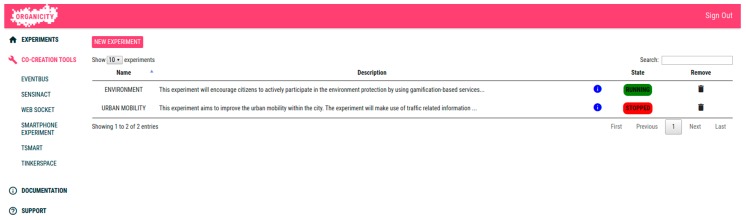
Experiment list within the main view of the EP.

Before starting the experiment, the experimenter creates an opportunistic communication experiment by means of the EP, providing its details (e.g., duration, area, etc.). For this purpose, he/she needs to be registered as an experimenter in the OC platform, so that, once he/she logs in the EP, is redirected to the main page of the EP, shown in Figure 10. This mail page provides quick access in the side bar to the co-creation tools that facilitate the development of new services and applications; to document tools and APIs necessary for the experiment development; and to provide supporting information, including links to the OrganiCity FAQ system or dedicated channels devoted to providing timely support to the experimenters of every component within the OC platform through the GitHub platform. 

The list of current experiments is shown in the main view, indicating whether they are ongoing or not, so that the experimenter can see the experimentation state at a glance. As can be seen, the portal provides the option to remove experiments, as well as to create new ones.

In the OrganiCity context, the experiment is given both spatial and temporal scopes. Moreover, for each experiment, there are a set of assets, or information sources that can be created/updated within the scope of the experiment or other ones that can be consumed. Apart from that, the EP can handle and configure the set of applications (e.g., mobile application) that belong to the experiment.

During the creation of an experiment, the experimenter indicates, along with other information, the experiment area, consisting of a set of regions, in which the experiment will take place. This area will be used as a first filter to select, by using some UDO functionalities, the assets used along the experiment.

**Figure 11:**
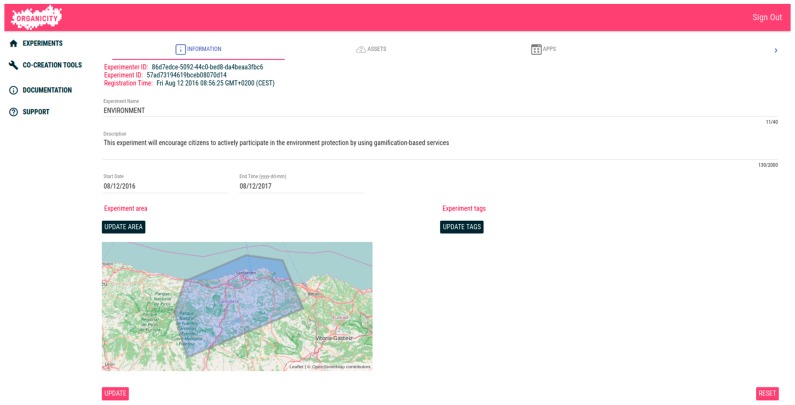
Opportunistic communication experiment details.

Once the experimenter has finished with the creation of this opportunistic communication experiment, he/she can select it from the list, and then its details are presented. Firstly, as can be seen in Figure 11, the information indicated during the experiment creation is displayed, allowing a user of the portal to modify it. By this way, an experimenter might modify the experiment settings during its lifecycle: extending or limiting the area of the experiment, modifying its timespan or editing the experiment tags to better indicate its aim.

Apart from the general experiment information, the EP also shows the different assets related to the experiment. In this regard, the portal differentiates between the assets created by the experiment itself (e.g., information gathered by experiment applications) from those existing assets that were selected to be used in the experiment. In the case of the opportunistic communication experimentation, it differentiates between those assets that act as relay nodes or as static nodes. 

Concerning the assets selected, the Experimenter Portal provides access to one of the UDO functionalities, which permits the search of assets under different criteria provided they are within the experiment area, which, as commented before, is the first filter to be applied. Once the assets are selected, they will be visible in the portal and their current availability.

On the other hand, concerning the assets created by the experiment (i.e., information collected during the opportunistic communication experiment), the portal also allows creating new assets by means of an editor, with a twofold purpose: first to simplify the definition of new information sources by a visual tool, and secondly to provide a template and useful insight to create assets via software. 

Furthermore, the Experimenter Portal also provides the functionality to manage applications to be used during the experiment. In the case of this experiment, the link where the android application can be downloaded is provided. Upon that registration of application to the experiment, a new application id belonging to this particular experiment will be assigned to the experimenter. The information about the experiment can be updated in the future as well. The created experiment is then advertised to potential participants through a community management tool. When a user finds the experiment on OrganiCity app and would like to join the experiment, the smartphone application registered to this particular experiment can be downloaded onto a user’s phone and starts running. The application uses the information, e.g., experiment ID and application ID, to send the data to the OppNet server and the measurements to the OC Experimenters site. 

Finally, the experimenter is able to configure the parameters from the Experimenter Portal that is used as the objective function that drives the routing and incentivisation protocols. Put simply, if a given phone has a choice of a number of phones in its locality to route data through, it makes the decision based on three aspects. Firstly, the phone that indicates it will more likely meet other phones is consequently more able to route data quickly. Secondly, a phone that has more energy is less likely to lose data as the phone will not die before it sends the data onwards. Given the incentivisation takes the cost of using the phone into account in terms of energy costs, the lower cost phone will also be chosen to minimize the cost of sending data around the network. A weighting parameter α, represents a notion of reliable communication, and when set to 0, the routing decision is purely a backpressure algorithm that does not take the energy left in devices into account. That is, it is solely dependent on the queue difference of the smart phones. When α is increased, more weight will be put on battery energy difference. The *selling* price also takes α into account as the use of energy is a cost. This is calculated using Qx(t) + α × (Bmax(t) − Bx(t)). The buyer pays this price, while the seller earns this price. In this function, Qx is the queue length for a device x. Bmax is the maximum battery level a device has. Bx is the current battery level of the device x. During the experiment, data is exchanged between phones and the incentivisation credits for each participant are calculated based on the price paid and earned. The phone initially receives data from static IoT devices. When a phone is near a free communications link (WiFi), it transmits data to the OC platform. Furthermore, α can also be updated dynamically during the experiment. That is, the application is able to retrieve this parameter from the Experimenter Portal and thus change the incentivisation scheme on the fly. 

### 4.2. Opportunistic Network Services Integration within the OC Experimenter Tier

In order to carry out the opportunistic communication experimentation, the OppNet framework is set up at OrganiCity sites. It builds a dynamic wireless communication “infrastructure” that can feed data to the OC experimenters site (in the future, this scenario can be exploited by cities like London, Santander or Aarhus to validate their own solutions). 

To be involved in the experiment, the OC participants that wish to contribute to this service have to install the OppNet app in their smartphones. The participating users are registered to the platform via the community management tool. In this experiment, the OppNet app provides the middleware for opportunistic communication experimentation that enables smartphones to collect data from IoT devices and subsequently feed it to OrganiCity. Overall, this component provides the major functionalities for the system for discovery and communication between the devices that form this opportunistic networking substrate.

In terms of communication, the following activities are carried out:

- Static IoT device to smartphone: discovery, connection and data transfer
- Smartphone to smartphone: discovery, connection and data transfer. Energy-aware Backpressure routing, a modified approach of selfish mules [24] is implemented for opportunistic data transfer between smartphones.
- Smartphone to OrganiCity: connection and data transfer through WiFi.

In order to facilitate the experiment monitoring, all devices used by participants within the experiment send a record of incentivisation credits to an OppNet server, which aggregates the information of all received user data. Such data will is sent to OC platform for further visualization periodically. The content of the data includes necessary authentication tokens and the accumulated rewards the users have received. The community management tool will then give feedback to the participants, based on the received reward data. This will be implemented in the form of a push notification in the community management service and the application will register for receiving such notifications. For instance, if a user gets a low reward, the community management tool will notify this user to move in to a specified area to have more social contacts, or have phone battery fully charged and have access to free WiFi more often.

**Figure 12:**
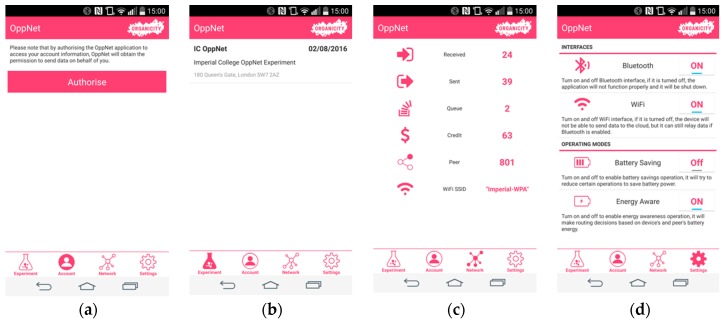
Screenshots from the OppNet smartphone application, (**a**) Authentication; (**b**) Experiments List; (**c**) Network; (**d**) Settings.

As mentioned previously, the OppNet application is currently implemented on the Android OS. Screenshots of the android app for various functionalities, as shown in Figure 12. Figure 12a illustrates the function for authorizing the use of this application for OrganiCity platform, and Figure 12b shows currently available experiments for participants to select. Figure 12c shows current network status, such as Bluetooth communication statistics, WiFi connection status and the rewards the participants have earned. Figure 12d shows the settings page, which not only allows users to turn on/off Bluetooth and WiFi, but also provides two other functions: battery saving and energy awareness. Battery saving provides a low battery operation mode for the application in order to not drain the battery too fast due to some battery hungry operations such as Bluetooth scanning. When the battery saving option is enabled, the Bluetooth scanning interval is increased, while the scanning duration is decreased, in order to consume less power. The energy awareness option is used to enable the context-aware Backpressure routing protocol, by considering the current battery level when making routing decisions. It makes the data more likely to flow from devices with low battery levels to ones with high battery levels, thus making sure that the network does not lose the data when a smartphone goes offline due to low battery.

### 4.3. Experimental Results

To demonstrate how an opportunistic connectivity service will be provided for OrganiCity with citizens’ participation, an experiment has been conducted at London city. Participants were recruited to take part in the experimentation based on their home location, where they carry out a majority of their activities i.e., work place or home. This experiment was carried out with 1–5 participants for the duration of three days. Various statistics collected and recorded during the experiment are shown in Table 1.

**Figure 13:**
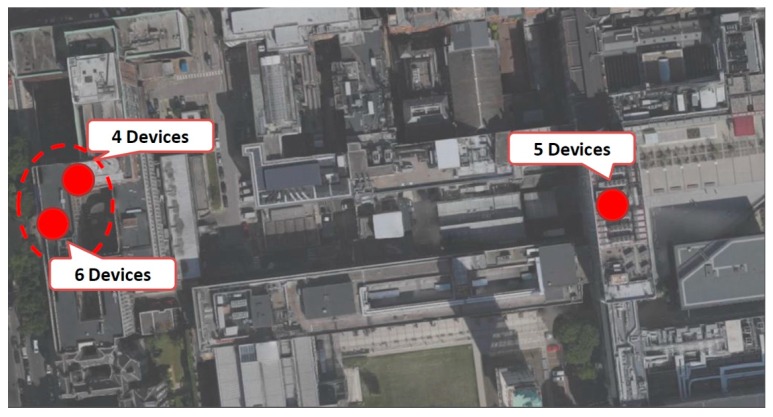
Geographical distribution of participants.

The spatial distribution of the participants is shown in Figure 13, which show the home location of the participants who took part in the experiment. The red dots represent the communities of the participants of the experiment based on their home locations. Such a social structure can be deduced by creating a contact graph from experimental results without the prior knowledge of exact locations of participants. 

During the experiment, all participants downloaded and installed OppNet application from the experimentation portal. On execution, OppNet app registers the volunteers to the experiment and starts collecting data from the sensing component. For this experiment, we measured luminosity data (in lux) generated from the available sensors on the smartphones of the participants. The data is then routed to other mobile devices taking part in the experiment until it is finally sent to the opportunistic communication server through WiFi in a multi-hop manner.

OppNet server gathered and processed all the sensing data sent from the mobile devices and fed the information to the Experimentation Portal. It also verified the integrity of the data and performed the checks if a smart device had permission to upload data for the experiment.

**Figure 14:**
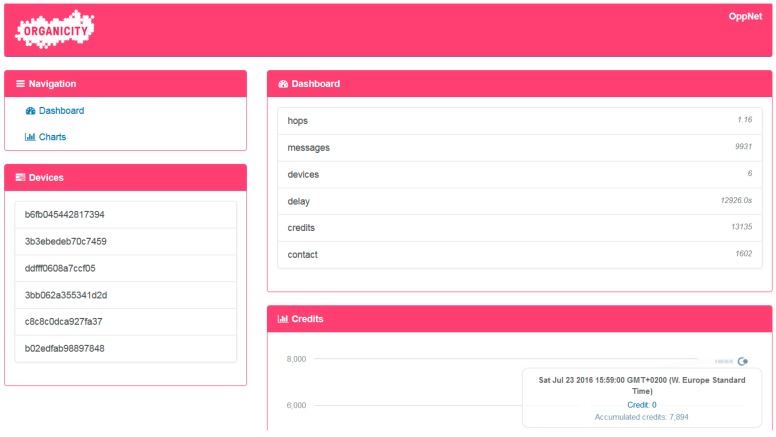
Screenshots of the OppNet visualization server.

From the Experimentation Portal, the experimenters are able to visualize and monitor the progress of the experiment as shown in Figure 14. The information about participants’ activity is monitored during the experiment through summary tables of the incentivization credits and contact information of the participants. This information provides better understanding of the interaction among all participants and helps to analyze mobility patterns and social structures.

The information about delay, packet drop and hop count is also available for experimenters to evaluate the performance of the opportunistic communication experiment. This data is presented in tables and graphs for a better understanding of the opportunistic communication system.

**Figure 15:**
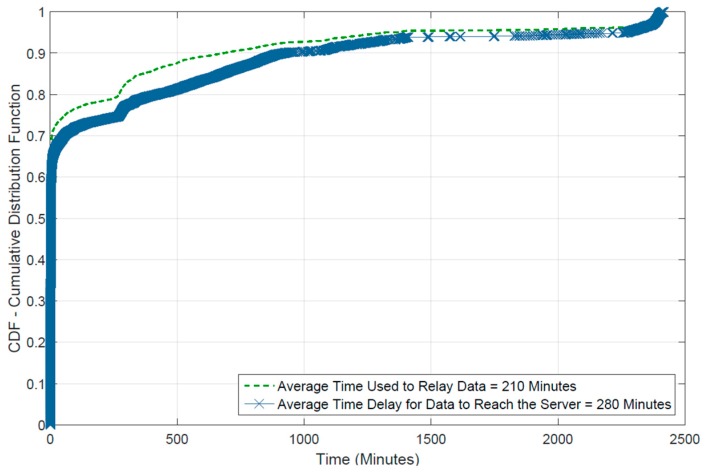
Time delay: single hop relay (blue) and end-to-end (green).

**Figure 16:**
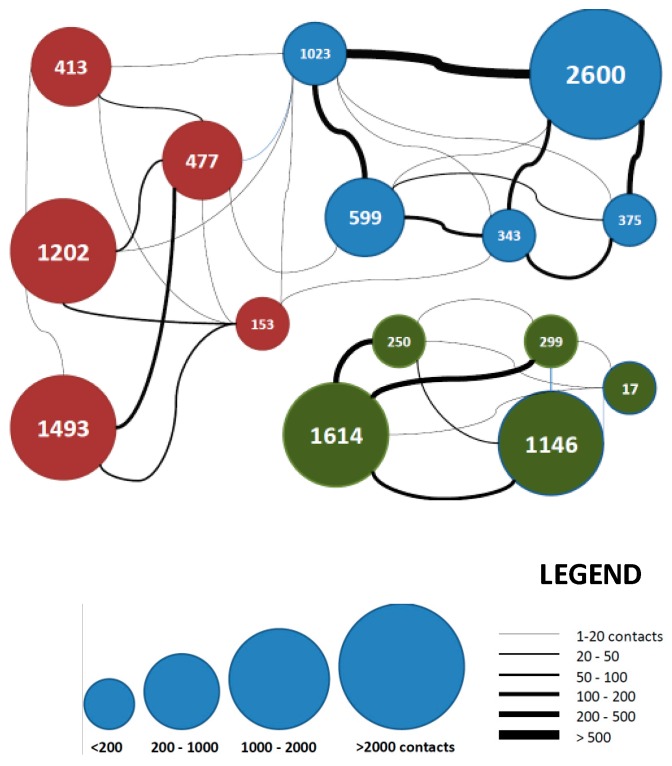
Contact graph of participating devices.

The final results from the experiment to measure end-to-end delay are shown as CDF plot in Figure 15. It shows that 70% of data was received in less than 10 min, which validates the feasibility of opportunistic communication for delay tolerant data. As discussed before, the participants can form different communities based on their locations. A contact graph between participants is shown in Figure 16 to reflect the social structure. Each participating device is shown as a bubble where color indicates the community, size of the bubble show WiFi availability during experiment and the numbers inside bubbles tell the count of relayed messages by a participant. Width of the connected lines between bubbles indicate the number of contacts between them. As it can be seen, three communities can be deduced from the contact data. Participants belonging to same community meet each other more often and they are joined by thicker lines in the graph.

Each bubble represents one participating device, color indicates community structure, bubble size for WiFi availability, the digits in the bubbles for number of relayed messages and width of lines connecting bubbles for contact times.

## 5. Conclusions

Smart cities are unique ecosystems in co-creating the cities of the future. After around a decade conceiving basic technologies to be deployed in the cities aiming at making them more efficient and sustainable, it is now the right time to go one step forward. In the same way that municipalities offer transportation, lighting or environmental services to their citizens, they have to provide facilities which enable citizens to improve their skills in interacting with the seamless technology surrounding them as well as enabling them to create new services and solutions aimed at improving life in the city. It is in this context that the OrganiCity platform represents a pioneering, holistic approach in designing, implementing and validating an EaaS facility relying on the federation of different city assets. The facility can be scaled up with the aggregation of more assets in terms of additional cities but also in terms of citizens wishing to expose their own ones. Furthermore, issues such as incentivisation and rewards play a key role in our approach. The platform has demonstrated it has sufficient flexibility to conceive and integrate a myriad of incentivisation policies aiming at engaging the city stakeholders with different roles depending on the type of experiment. Last but not least, the trustworthiness and reliability of the exposed data can also be handled by data annotation tools explicitly developed for this purpose. 

Looking at the future, there are several important aspects to consider. One refers to the conception of solutions linked to the sustainability of such EaaS platforms. Hence, it is needed to valorize facilities such as OrganiCity in the same way that citizens do with traditional city services. Frameworks such the Digital Single Market can help in reaching such an objective. 

## Figures and Tables

**Table 1:** OppNet datasets.

Data Entries	Participants	Contacts	Experiment Duration
9016	15	12,004	50 h

## Abbreviations

The following abbreviations are used in this manuscript:

4G	4th Generation
AAA	Authentication, Authorization, and Accounting
API	Application Programming Interface
CDF	Cumulative Distribution Function
D2D	Devide to Device
DTN	Data Transmission Network
EaaS	Experimentation as a Service
EM	Experimentation Management
EMS	Experimentation Management Services
EP	Experimenters Portal
FAQ	Frequently Asked Question
FI	Future Internet
FM	Facility Management
FMP	Facility Management Portañ
FMS	Facility Management Services
ICT	Information and Communication Technologies
IoT	Internet of Things
KPI	Key Performance Indicators
MV	Model View Controller
OC	OrganiCity	
OppNet	Opportunistic Network
QoS	Quality of Service
REST	Representational State Transfer
RFID	Radio Frequency Identification
UDO	Urban Data Observatory
UI	User Interface	
UX	User Experience
